# An update of anti-viral treatment of COVID-19

**DOI:** 10.3906/sag-2106-250

**Published:** 2021-12-17

**Authors:** Serap ŞİMŞEK YAVUZ, İpek KOMŞUOĞLU ÇELİKYURT

**Affiliations:** 1 Department of Infectious Disease and Clinical Microbiology, İstanbul Faculty of Medicine, İstanbul University, İstanbul Turkey; 2 Department of Pharmacology, Faculty of Medicine,Kocaeli University, Kocaeli Turkey

**Keywords:** SARS-CoV-2, COVID-19, antiviral, treatment

## Abstract

**Background/aim:**

Currently there is not an effective antiviral treatment for COVID-19, but a large number of drugs have been evaluated since the beginning of the pandemic, and many of them have been used for the treatment of COVID-19 despite the preliminary or conflicting results of the clinical trials. We aimed to review and summarize all of the current knowledge on the antivirals for COVID-19.

**Results:**

There are 2 main drug groups for SARS-CoV-2: agents that target proteins or RNA of the virus or interfere with proteins or biological processes in the host that support the virus. The main drug groups include inhibitors of viral entry into the human cell (convalescent plasma, monoclonal antibodies, nanobodies, mini proteins, human soluble ACE-2, camostat, dutasteride, proxalutamide, bromhexin, hydroxychloroquine, umifenovir nitazoxanid, niclosamide, lactoferrin), inhibitors of viral proteases (lopinavir/ritonavir, PF-07321332, PF-07304814, GC376), inhibitors of viral RNA (remdesivir, favipiravir, molnupiravir, AT-527, merimepodib, PTC299), inhibitors of host proteins supporting virus (plitidepsin, fluvoxamine, ivermectin), and agents supporting host natural immunity (Interferons).

**Conclusion:**

When taking into account the results of all the available laboratory and clinical trials on the subject, monoclonal antibodies seem to be the most effective treatment for COVID-19 at the moment, and high-titer convalescent plasma also could be effective when administered during the early phase of the disease. As lopinavir/ritonavir, hydroxychloroquine, merimepodib, and umifenovir were found to be ineffective in RCTs, they should not be used. Additional studies are needed to define the role of remdesivir, favipiravir, interferons, ivermectin, dutasteride, proxulutamide, fluvoxamine, bromhexine, nitazoxanide, and niclosamid in the treatment of COVID-19. Finally, the results of phase trials are waited to learn whether or not the newer agents such as molnupiravir, PF-07321332, PF-07304814, plitidepsin and AT-527 are effective in the treatment of COVID-19.

## 1. Introduction 

Although 18 months have been passed since the beginning of COVID-19 pandemic, an effective antiviral treatment still has yet to be found. The big achievement of finding highly effective vaccines was not repeated in the case of antiviral treatment. It is obvious that the success of mRNA vaccines mainly relies on nearly 30 years’ hard work of numerous scientists [1]. Despite warnings of scientists and policymakers in the field to be prepared for the next pandemic and develop and stockpile drugs that target a wide range of viral pathogens since the 2003 SARS epidemic, pharmaceutical companies, and researchers ignored the warns. 

After the SARS outbreak was taken under control, all of the antiviral studies terminated. If those researches had been completed, we would have had something in the stockpile for the treatment of COVID-19 now [2]. But with the help of new huge investments, it is hoped that effective antivirals could be found by the end of 2021Economic Times. Anti-Covid tablets by 2021 end? Fauci says US to spend $3.2bn for COVID antiviral pills. Website https://economictimes.indiatimes.com/news/international/world-news/anti-covid-tablets-by-2021-end-fauci-says-us-to-spend-3-2bn-for-covid-antiviral-pills/videoshow/83627082.cms [accessed 19 June 2021].. 

Early efforts to identify effective antiviral for COVID-19 have mainly focused on drug repurposing screens to identify drugs with antiviral activity against SARS-CoV-2 in cell cultures. But the cell types in the cultures were not always appropriate to predict the physiological and pathological events in the human cells. For example, a deeper understanding of the viral entry pathways revealed that agents affect only endosomal pathways but not fusional ones, for example hydroxychloroquine would be ineffective for the treatment of COVID-19. In addition to understanding the viral entry pathways of SARS-CoV-2 to different cell types, consideration also should be given to the degree of nucleotide prodrug activation observed in *the in vitro* screening cell types [3]. 

The majority of COVID-19 patients will recover without any therapy. However, initiating therapy after patients are severely ill could not be effective as it is known that antiviral therapy is mostly useful when initiated earlier during the course of the disease. It is logical to start antiviral treatment immediately for COVID-19 patients especially in whom at high risk of severe disease [4]. 

In this review, we aimed to summarize the current knowledge on the antiviral treatment of COVID-19. Investigated antivirals for SARS-CoV-2 can be divided into 2 groups: agents that target proteins or RNA of the virus; S protein, viral proteases (nonstructural protein (NSP)-3 and NSP-5), viral RNA dependent RNA polymerase (NSP-12) are the main viral targets. Agents that target host proteins are host proteases, which can help entry of the virus into the cell (Angiotensin converting enzyme-2 (ACE-2), transmembrane protease serine 2 (TMPRSS2), furin, catepsin-L), heparin sulfate proteogylicans (HSPGs) that promote the viral cell attachment, eukaryotic translation proteins (translation initiation factor 4A (eIF4A), translation elongation factor 1a (eEF1A), endosplasmic reticulum chaperon protein (S1R, etc.)), transcription machineries (inosine monophospate dehydrogenase, dihydroorarate dehydrogenase, etc.), and host nuclear importer of viral proteins ( IMPα/β1) [2] (Figure).

**Figure:**
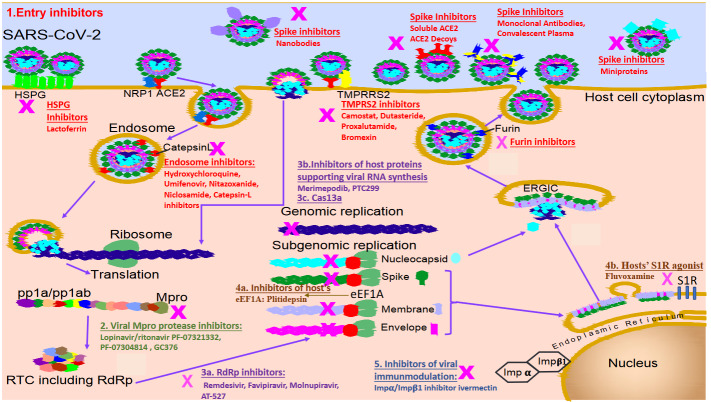
Drugs targeting various stages of life cycle of SARS-CoV-2.

Those are the drug groups that either laboratory or clinical studies are going to find as an effective antiviral against SARS-CoV-2**:**

1) Inhibitors of viral entry into the human cell: 

a. Inhibitors of S protein: Convalescent plasma, monoclonal antibodies, nano bodies, miniproteins, human soluble ACE-2.

b. Inhibitors of fusional entry:

i. TMPRSS2 inhibitors (camostat, nafamostat, gabexat, dutasteride, proxalutamide, bromhexin, nitazoxanid, niclosamide),

c. Inhibitors of endosomal entry: Catepsin L inhibitors (teicoplanin, SSAA09E1, K1777); furin inhibitors (dec-RVKR-cmk), NIP1 inhibitors (EG00229), hydroxychloroquine, nitazoxanide, niclozamide, umifenovir,

d. HSPG inhibitors (lactoferrin). 

2) Inhibitors of viral proteases: inhibitors of viral main protease (Mpro) (lopinavir/ritonavir, PF-07321332, PF-07304814, GC376); inhibitors of viral papain-like protease (PL pro). 

3) Inhibitors of viral RNA: inhibitors of RNA dependent RNA polymerase (RdRp) (remdesivir, favipiravir, molnupiravir, AT-527), inhibitors of host proteins supporting viral RNA synthesis (inhibitor of inosine monophospate dehydrogenase (merimepodib), inhibitor of dihydroorarate dehydrogenase (PTC299). 

4) Inhibitors of host proteins supporting viral protein synthesis: inhibitors of eEF1A (plitidepsin); S1R agonists (fluvoxamin).

5) Inhibitors of viral immunomodulation: Inhibitors of host importin α/β (ivermectin).

6) Agents supporting host natural immunity: interferons. 

## 2. Inhibitors of virus entry into the human cell 

The attachment and entry of SARS-COV-2 into the human cells is the first step in the COVID-19 pathogenesis. SARS-CoV-2 enters the host cells by binding its spike (S) protein to the cellular ACE2. In recent studies, cell surface HSPGs were reported to be a necessary cofactor for ACE2 mediated SARS-CoV-2 entry [5,6]. The virion enters through endocytosis and/or direct fusion of cell and viral membranes. The SARS-CoV-2 spike (S) glycoprotein is a transmembrane homotrimer. The S protein is cleaved by various cellular proteases (e.g., TMPRSS2, furin, catepsin L, etc) into two subunits, S1 and S2, which is named as priming process, and S2 functions as fusion peptide [7]. As the entry of SARS-CoV-2 into host cells is critical for infection, it becomes an extremely attractive therapeutic interventional step [8]. 

Viral entry inhibitors target either S protein (convalescent plasma, monoclonal antibodies, nanobodies, miniproteins and soluble human ACE-2) or host ACE-2 receptor, or host proteins including proteases and HSPGs that help viral entry (Figure). 

### 2.1. Inhibitors of S protein

#### 2.1.1. Convalescent plasma 

Given the lack of effective treatment of COVID-19, classical interventions like convalescent plasma (CP) have remerged as treatment options. CP is a strategy of passive immunization and has been used in prevention and treatment of so many infectious diseases since early 20th century. The CP is obtained using apheresis in survivors with prior COVID-19 in whom neutralizan antibodies (NAbs) against the SARS-CoV-2 are developed. The efficacy of this therapy has been shown to be associated with the concentration of NAbs directed to the receptor-binding domain (S1-RBD) and N-terminal domains (S1-NTD) of S protein. Those NAbs inhibit the entry of SARS-CoV-2 into the host cell and limit viral amplification [9]. 

Several retrospective observational studies in 2020 suggested a beneficial role of CP for patients hospitalized with severe COVID-19, and these initial reports led to Emergency Use Authorization (EUA) of CP all over the World [10, 11]. But following randomized controlled trials (RCT)s did not confirm the positive findings of those preliminary observational studies [12]. 

In a metaanalysis of randomized clinical trials (1060 patients) with COVID-19 treated with CP versus control, it was concluded that treatment with CP compared with placebo or standard of care was not significantly associated with a decrease in all-cause mortality or with any benefit for other clinical outcomes [13]. 

But in a recent randomized, double-blinded, placebo-controlled trial of 160 older adult patients within 72 h after the onset of mild COVID-19 symptoms, early administration of high-titer CP against SARS-CoV-2 reduced the progression of COVID-19 significantly by a relative risk of 0.52 (95% confidence interval [CI], 0.29 to 0.94; p = 0.03) [14]. 

Also, in a retrospective study of 3082 patients hospitalized with COVID-19, transfusion of plasma with higher anti–SARS-CoV-2 IgG antibody levels were associated with a lower risk of death than transfusion of plasma with lower antibody levels with a relative risk of 0.66 (95% CI, 0.48 to 0.91) among patients who had not received mechanical ventilation before transfusion [15]. 

These studies suggested that CP against COVID-19 work better when administered early (first 72 h of symptoms, etc.) in the course of the illness and a dose-dependent IgG effect in CP infusions. Taking into account of extremely higher NABs titers among mRNA immunized persons [16], it will be quite possible that CP of those people could contribute to building a cheap and effective therapeutic option in the future with the help of additional RCTs. 

In conclusion, high-titer CP given within 72 h of symptom onset to high-risk adult outpatients (including ≥65 years, obesity, with comorbidities diabetes mellitus, hypertension, chronic obstructive lung disease, cardiovascular disease, chronic renal failure) with mild illness seems to have efficacy in reducing the risk of progression to severe disease. However, high-titer CP therapy is still investigational and should be administered in the context of a clinical trial if possible. 

#### 2.1.2. Monoclonal antibodies

In the case of SARS-CoV-2, S glycoprotein is the target of neutralizing antibodies [17]. 

In RCTs, bamlanivimab, bamlanivimab-etesevimab, casirivimab-imdevimab, and sotrovimab (VIR-7831) reduced the rate of hospitalization plus dead (visit to an emergency department for bamlanivimab) by 61%, 87%, 72%, and 86%, respectively [18,19] Investor/Lilly. Lilly’s bamlanivimab and etesevimab together reduced hospitalizations and death in Phase 3 trial for early COVID-19 Website https://investor.lilly.com/news-releases/news-release-details/lillys-bamlanivimab-and-etesevimab-together-reduced [accessed 16 June 2021]., Gupta A, Gonzalez-Rojas Y, Juarez E, Casal MC, Moya J, at al. Covid-19 Treatment With SARS-CoV-2 Neutralizing Antibody Sotrovimab. medRxiv preprint 2021; doi: https://doi.org/10.1101/2021.05.27.21257096[accessed 16 June 2021]. among outpatients. In the global phase 2/3 trial of regdenvimab, it was reduced the progression rate to severe COVID-19 by 54% for patients with mild-to-moderate symptoms [17]. But, in an interim analysis of an RCT, bamlivimab was found to be ineffective among hospitalized patients with COVID-19, and the study was stopped early for futility [20]. 

In the RECOVERY’s monoclonal antibody combination of casirivimab and imdevimab (REGEN-COV) trial, 9785 patients hospitalised with COVID-19 were randomly given usual care plus REGEN-COV or usual care alone, including 3153 (32%) seronegative and 5272 (54%) seropositive patients. They found that, in patients hospitalised with COVID-19, the monoclonal antibody REGEN-COV combination reduced 28-day mortality among patients who were seronegative at baseline (rate ratio 0.80; 95% CI 0.70–0.91; 43 p = 0·0010) RECOVERY Collaborative Group. Casirivimab and imdevimab in patients admitted to 4 hospital with COVID-19 (RECOVERY): a randomised, controlled, open-label, platform trial. medRxiv preprint 2021; doi: https://doi.org/10.1101/2021.06.15.21258542. [accessed 16 June 2021]..

Several mAbs received EUAs all over the world including, USA, Europe, South Korea, and India. As no dose response was observed in most of those clinical trials, it was thought that mAbs likely overdosed. Lower doses could be administered by more easy routes such as intramuscular or subcutaneous, and the shift to these different routes is underway and will possibly contribute to facilitated and larger access to these mAb [17]. In a RCT, subcutaneous (SC) REGEN-COV 1200mg was compared with placebo in SARS-CoV-2-positive cases. REGEN-COV 1200mg SC significantly prevented progression from asymptomatic to symptomatic disease compared with placebo (31.5% relative risk reduction; 29/100 (29.0%) versus 44/104 (42.3%), respectively; p = 0.0380) O’Brien MP, Forleo-Neto E, Sarkar E, Isa F, Hou P, et al. Subcutaneous REGEN-COV Antibody Combination in Early SARS-CoV-2 Infection. medRxiv preprint 2021; doi: https://doi.org/10.1101/2021.06.14.21258569. [accessed 16 June 2021].. 

After getting encouraging results from the RCTs, COVID-19 treatment guidelines recommended bamlanivimab/etesevimab or casirivimab/imdevimab for ambulatory patients with mild to moderate COVID-19 (not requiring supplemental oxygen**) **and at high risk for progression to severe disease**. **These risk factors include older age (≥65 years), obesity, pregnancy, chronic kidney disease, diabetes mellitus, immunosuppression, cardiovascular diseases, chronic lung disease, neurological development disorders, sickle cell disease, and other medically complex conditions Bhimraj A, Morgan RL, Shumaker AH, Lavergne V, Baden L, et al. Infectious Diseases Society of America Guidelines on the Treatment and Management of Patients with COVID-19. Infectious Diseases Society of America 2021; Version 4.3.0. Website https://www.idsociety.org/practice-guideline/covid-19-guideline-treatment-and-management/. [accessed 16 June 2021]. 

As evidence from CP treatment show that benefit is maximized with early administration, mABs treatment should be given as soon as possible after the diagnosis and within seven days of symptom onset. 

All mAbs authorized or in development are directed to the RBD, which interacts with the target receptor ACE2 and, as a result, could be susceptible to mutation of viral RBD. Immune-evading SARS-CoV-2 variants have been emerging from the beginning of the pandemic and may potentially continue to emerge with implications for mAb therapeutics. As a result, it is recommended that local variant susceptibility should be considered in the choice of the most appropriate neutralizing antibody therapy. In April 2021, Eli Lilly requested revocation of the EUA for bamlanivimab, because of an increase in variants resistant to bamlanivimab monotherapy. In any case, emergence of resistance to mAbs should be monitored for all variants [17]. Both high-titer CP and mAbs may be more important and efficient for subgroup of immunocompromised patients with B-cell depletion.

#### 2.1.3. Nanobodies

The camelid family, which includes lamas and camels, produces only heavy chain antibodies that are composed of two heavy chains. Nbs have the ability to function as an independent antigen-binding domain with similar affinity as a conventional IgG. Nbs have been successfully humanized without significant alteration of biophysical properties. Caplacizumab, the first-in-class nanobody was approved recently for the treatment of thrombotic thrombocytopenic purpura [21], which boosters the therapeutic potential of Nb derivatives. Nbs that bind to the SARS-CoV-2 RBD and block the ACE2 interaction could be an attractive therapeutic option for the prevention and treatment of COVID-19. Nbs that primarily target the RBD of the SARS-CoV-2 S glycoprotein for virus neutralization were developed recently [22, 23, 24]. Highly selected Nbs and the multivalent forms obtain high neutralization potency comparable to, or even better than, some of the most successful SARS-CoV-2 neutralizing mAbs. One of the aerosolized Nbs is Pittsburgh inhalable Nanobody 21 (PIN-21), which efficiently blocked SARS-CoV-2 infectivity at low doses in vitro [25]. Aerosol and intranasal delivery of PIN-21 was also shown to be effective to prevent and treat COVID-19 [26]. But before moving into human clinical trials, further preclinical analyzes are needed. 

#### 2.1.4. Miniproteins 

Miniproteins, each with about 60 amino acids, are produced to block protein-protein interactions. Recently, miniproteins that can bind tightly to the SARS-COV-2’ S protein and block it from attaching to the ACE2 receptor was described [27]. Potential advantages of these miniproteins over antibodies are as follows: resistance to viral mutational escapes, not requiring a cold storage condition, possibility of a gel or aerosol formulation, and easier production steps [27,28]. But before clinical studies, miniproteins also need to be evaluated properly in the preclinical studies. 

#### 2.1.5. Human soluble ACE-2 

Human recombinant soluble ACE2 (hrsACE2) theoretically should be of benefit in COVID-19 by binding S protein and thereby neutralizing SARS-CoV-2 and by minimizing injury to multiple organs because of renin–angiotensin system hyperactivation and increased angiotensin II concentrations. In a phase 2 clinical trial, hrsACE2 has been tested in patients with acute respiratory distress syndrome (ARDS) and was shown to has an acceptable safety profile [29]. It was also shown to reduce SARS-CoV-2 load by a factor of 1000–5000 in-vitro cell-culture [30]. Finally, a case of severe COVID-19 was treated successfully with hrsACE2 [31]. 

A phase 2 clinical trial of hrsACE2 compared to placebo (NCT04335136) in 178 patients with severe COVID-19 was completed (NCT04335136), and the results were shared via press release: It was reported that all-cause mortality or invasive mechanical ventilation was lower among patients treated with hrsACE2 compared to placebo, but the difference was not statisticaly significant. HrsACE2 also demonstrated a positive impact on biomarkers of the renin angiotensin system Apeiron Biologics. APEIRON’s APN01 shows clinical benefits for severely ill COVID-19 patients in phase 2 trial. Website https://www.apeiron-biologics.com/apeirons-apn01-shows-clinical-benefits-for-severely-ill-covid-19-patients-in-phase-2-trial/[accessed 16 June 2021]..

#### 2.1.6. ACE2 receptor trap molecules 

ACE2-like molecules like CTC-445.2d can be used as decoys for the S protein and could draw SARS-CoV-2 away from cells. CTC-445.2d which developed recently is a miniprotein that mimics ACE2, binds firmly to spike and neutralize SARS-CoV-2. It can simultaneously bind to all three RBDs of a single spike protein. CTC-445.2d potently neutralized SARS-CoV-2 infection of cells in vitro, and a single intranasal prophylactic dose of decoy protected Syrian hamsters from a subsequent lethal SARS-CoV-2 challenge [32]. 

Another receptor trap molecule described by other researchers was also neutralized 

SARS-CoV-2 infections as effectively as high-affinity antibodies isolated from convalescent patients and also binds S proteins of other human coronaviruses. As ACE2 receptor traps have large binding interfaces, they are able to block the entire receptor binding interface, which limits the impact of viral escape mutations [33]. 

### 2.2. Inhibitors of fusional entry of virus

Host peptidases (furin, TMMPRS2, catepsin L) and some host proteins (NRP1) are critical for SARS-CoV-2 entry and act either as receptors for the attachment of the virion S protein to the target cell (like ACE2 and NRP1) or as facilitators of virion envelope fusion with the target cell membrane (like furin, TMPPRS2, and catepsin L). 

SARS-CoV-2 entry into host cells takes place either by receptor-mediated endocytosis or by membrane fusion. The binding of SARS-CoV-2 to the cellular receptor ACE2 can result in virion uptake into endosomes. The S protein is activated by cysteine peptidase cathepsin L in endolysosomes. In membrane fusion, precleavage of the spike at the polybasic S1/S2 sites by furin protease during viral egress is needed. Studies with SARS-CoV-2 show that this cleavage promotes subsequent virus infection. After that processing, TMPRSS2 cleaves spike at S2ʹ and facilitates early entry at or near the cell surface, as opposed to late entry through the endosome [34,35,36]. This route especially important for lung cells, which fail to express robust levels of cathepsin L. Furin cleavage at the S1-S2 junction also exposes the C-end rule peptide on SARS-CoV-2 S1 and allows binding to neuropilin-1 (NRP1), which may facilitate the entry of the SARS-CoV-2 into host cells and may act as a host cell mediator that can increase the infectivity and may, thus, contribute to the tissue/ organ tropism of this coronavirus [37,38]. Either way, the RNA genetic material of the virus is released, and the late stage of the life cycle subsequently takes place by RNA replication (Figure).

That’s why both host proteases furin and TMPRSS2 and host protein NRP1 are regarded as a potential drug targets [39]. Whilst inhibition of TMPRSS2 protease activity would not prevent infection via the endosome, using this pathway is detrimental to virus replication in airway cells [40]. 

#### 2.2.1.TMPRRS2 inhbitors

Camostate mesilate, nafomastat: Camostate mesilate originally developed in the 1980s in Japan and licensed for the treatment of chronic pancreatitis is an inhibitor of TMPRSS2. It was shown to block SARS-CoV-2 entry into lung cells in vitro [39] and to be a potent antiviral agent against SARS-CoV in vivo [41]. In a case-series of severe COVID-19 patients, camostat showed a clinical benefit in reducing the SOFA score [42]. But in an RCT of 137 patients hospitalized with confirmed SARS-CoV-2 infection, comparing the placebo, camostat mesilate 200 mg three times daily for 5 days did not reduce the time regarding clinical improvement and mortality [43]. Another clinically approved TMPRSS2 inhibitor, nafamostat, requires intravenous dosing and has also shown effect SARS-CoV-*2 *in vitro [39], but there isn’t any clinical study about the effectiveness of nafamostat for treating COVID-19 at the moment. 

As camostate mesilate or other TMPRSS2 inhibitors administered in higher doses or during the very early phase of COVID-19 might be effective in lowering the risk of disease progression, additional studies are needed.

##### 2.2.1.1.Antiandrogens (dutasteride, proxolutamide)

Considering that the activated androgen receptor regulates transcription of *TMPRSS2* gene, androgen hormone receptors signaling antagonists could be explored as treatment strategies against COVID-19 for their role in downregulating TMPRSS2. In this regard, it was assumed that men receiving androgen receptor signaling or androgen inhibitors for prostate cancer, prostate hyperplasia, or androgenic alopecia could have reduced risk of SARS-CoV-2 infection or severity [44]. Protection from more severe COVID-19 with the use of antiandrogens have shown in some observational studies [45].

In an RCT of 77 outpatients with mild/moderate confirmed COVID-19, 5-alpha-reductase inhibitor dutasteride, which is commonly prescribed antiandrogens for androgenic alopecia or benign prostatic hyperplasia was found to be related to higher virologic response, clinical recovery rate, and mean oxygen saturation compared to the placebo group on the seventh day of treatment [46].

An androgen receptor signaling inhibitor proxalutamide was also investigated in 3 RCTs for its effectiveness in treating COVID-19. In the first RCT, the rate of hospitalization and requiring mechanical ventilation were significantly lower in men treated with proxalutamide compared to standard of care among 214 mild/moderate confirmed COVID-19 male outpatients Cadegiani FA, McCoy J, Wambier CG, et al. Proxalutamide (GT0918) Reduces the Rate of Hospitalization and Death in COVID-19 Male Patients: A Randomized Double-Blinded Placebo-Controlled Trial.Research Square Preprint 2020. DOI: 10.21203/rs.3.rs-135303/v1. [accessed 16 June 2021].. 

 In the second RCT which included 236 mild to moderate confirmed COVID-19 patients, viral clearance rate on day 7 and clinical remission time was significantly lower in patients treated proxalutamide versus placebo [47].

In the third RCT, the results of which have not been published yet, comparing the placebo proxulatamide administration in the first 48 h of symptoms was found to be related significantly with quicker clinical remission, lower rate of hospitalization, and mechanical ventilation requirement and mortality among 588 confirmed COVID-19 outpatients Biospace, Kintor Pharmaceutical Announces Results from Investigator-Initiated Brazil Trial Demonstrating 92% Reduction in Mortality in Hospitalized COVID-19 Patients, March, 11, 2021. Website https://www.biospace.com/article/releases/kintor-pharmaceutical-announces-results-from-investigator-initiated-brazil-trial-demonstrating-92-percent-reduction-in-mortality-in-hospitalized-covid-19-patients/[accessed 16 June 2021].. 

But those encouraging findings should be confirmed with additional, larger scale RCTs. 

##### 2.2.1.2.Bromhexine

Bromhexine is another potent inhibitor of TMPRSS2. In a small open-label trial, a total of 78 hospitalized patients with probable COVID-19 were randomized to bromhexine and standard treatment arm; there was a significant reduction in ICU admissions and death in the bromhexine treated group compared to the standard group [48].

 But in another open label RCT of 100 hospitalized confirmed COVID-19 patients, there were no differences observed in the clinical improvement time, the mean intensive care unit stay, or risk of death by day 28 between the bromhexine and standard treatment arms [49].

Finally, in another RCT, bromhexine was found to be effective for protecting sypmtomatic COVID-19 among 50 healthcare workers, the results of the study have not peer reviewed yet Mikhaylov EN, Lyubimtseva TA, Vakhrushev AD, Stepanov D, Lebedev DS, , et al. Bromhexine Hydrochloride Prophylaxis of COVID-19 for Medical Personnel: 2 A Randomized Open-Label Study. medRxiv preprint 2021; doi:https://doi.org/10.1101/2021.03.03.21252855; [accessed 16 June 2021].. 

As the results of published studies are conflicting, more and larger scale RCTs are needed to define the place of bromhexin in COVID-19 treatment. 

### 2.3. Inhibitors of endosomal entry

#### 2.3.1. Hydroxychloroquine

Hydroxychloroquine (HCQ) is an aminoquinoline, which has been used to treat malaria and autoimmune diseases for over 50 years. Despite preliminary in vitro studies in kidney-derived Vero E6 cells reported that HCQ is effective against SARS-CoV-2, probably by inhibiting viral transport in endosomes by alkalinizing the intra-organel compartment; later studies in the TMPRSS2-expressing human lung cell line Calu-3 did not replicate these results. This was because, instead of endosomal pathway that HCQ inhibits, SARS-CoV-2 primarly uses fusion pathway in which TMPRSS2 activates it for entry into lung cells [50].

HCQ has been the most studied drug for the treatment and prevention of COVID-19 since the beginning of the pandemic and more than 120 clinical RCTs using HCQ were registered in the trial registries [51].

Unfortunately, in a metaanalysis of 14 unpublished (including1308 patients) and 14 published (including 9011 patients) RCTs, HCQ was found to be associated with increased mortality in COVID-19 patients with a combined OR of 1.11 (95% CI: 1.02–1.20; I² = 0%) for all-cause mortality and no subgroup effects were found [52].

Additionally, in another metaanalysis of six RCTs of prophylactic hydroxychloroquine usage (n = 6059 participants), it was found that compared with standard care or placebo, HCQ has no effect on suspected, probable, or laboratory-confirmed SARS-CoV-2 infection or does not reduce the risk of laboratory-confirmed SARS-CoV-2 infection [53]. 

As a result, current guidelines recommend against HCQ among hospitalized patients with COVID-19 with a strong recommendation and moderate certainty of evidence^6^. 

#### 2.3.2. Umifenovir

Umifenovir is a broad-spectrum antiviral agent which could effectively inhibit the fusion of virus with host cells and is already licensed for prophylaxis and treatment of

influenza. Previous research has revealed that umifenovir is an efficient inhibitor of SARS-CoV-2 in vitro. Nevertheless, little is known about the actual clinical efficacy of umifenovir *in* *vivo*.

Umifenovir is an antiviral agent which could inhibit the fusion of the virus with host cells and is already licensed for prophylaxis and treatment of influenza in Russia and China. It was shown that umifenovir inhibits SARS-CoV-2 in vitro with a 50% effective concentration (EC50) of 4.11 μM [54]. 

But in a metaanalysis of 12 clinical studies with 1052 patients, compared with the control group, umifenovir was not associated with lower risk of admission to intensive care unit or mechanical ventilation or death (RR:1.20; 95% CI: 0.61 to 2.37), a lower rate of symptoms alleviation on day 7, or lower hospital length of stay (MD: 1.34; 95% CI: –2.08 to 4.76) [55]. 

As a result, there is no evidence to support the use of umifenovir for patients with COVID-19.

#### 2.3.3.Nitazoxanide

Nitazoxanide, a small-molecule anti-protozoal drug is currently recommended for treating diarrhea caused by *Cryptosporidium* spp. or *Giardia* spp. Nitazoxanide targets against numerous points of SARS-CoV-2 life cycle including an endosomal and fusional entry into the host cells [56]. In the analysis of the key secondary endpoint, treatment with NT-300 was associated with an 85% (0.5% of NT-300-treated patients versus 3.6% of patients treated with placebo) reduction in the progression to severe illness Romark, 2021. Romark announces ınıtıal results of phase 3 clınıcal trıal of nt-300 tablets for the treatment of covıd-19. Website https://www.romark.com/romark-announces-initial-results-of-phase-3-clinical-trial-of-nt-300-tablets-for-the-treatment-of-covid-19/[accessed 16 June 2021].. The number of clinical trials was registered using nitazoxanide as the only drug or in combination with other antivirals for the treatment of patients with COVID-19 (NCT04486313, NCT04552483, NCT04348409), and results will be released soon. 

#### 2.3.4. Niclosamide

Niclosamide, which has been used to treat intestinal worms, also exhibits highly potent activity against SARS-CoV-2 in a Vero cell and human airway infection model [57] Weiss A, Touret[accessed 16 June 2021]. F, Baronti C, et al. Niclosamide shows strong antiviral activity in a human airway model of SARS-CoV-2 infection and a conserved potency against the UK B.1.1.7 and SA B.1.351 variant. bioRxiv 2021. DOI: 10.1101/2021.04.26.441457. [accessed 16 June 2021]. and inhibits SARS-CoV2 entry by blocking internalization through pH-dependent endocytic pathway Prabhakara C,  Godbole R, Sil P, Jahnavi S, Zanten TS,  et al. Niclosamide inhibits SARS-CoV2 entry by blocking internalization through pH-dependent CLIC/GEEC endocytic pathway. bioRxiv preprint 2020; doi: https://doi.org/10.1101/2020.12.16.422529; [accessed 16 June 2021].. The novel formulation demonstrates potent in vitro and in vivo activity against SARS-CoV-2 [58]. 

Another investigator from Denmark also developed a formulation of niclosamide optimized for inhalation and intranasal application called UNI91104, which is well-tolerated in healthy volunteers in phase1 clinical study [59]. 

Inhaler and nasal formulation of niclosamide are promising candidates for the treatment of viral respiratory infections such as COVID19. Further phase 1 and other clinical trials are being planned [59]. 

### 2.4. Heparan sulfate proteoglycan inhibitors: lactoferrin

It was shown in recent studies that in addition to ACE2, heparan sulfate proteoglycans (HSPGs) also has an important role in SARS-CoV-2 cell attachment [6, 59]. 

geLactoferrin showed a broad-spectrum antiviral activity against SARS-CoV-2, HCoV-OC43, HCoV-NL63, and HCoV-229E in cell culture via binding HSPGs and blocking viral attachment to the host cell. In cell culture, combination lactoferrin with remdesivir was found to have a synergistic effect against SARS-CoV-2. [60]. Further studies are needed to define the place of lactoferrin in the treatment of COVID-19. 

## 3. Inhibitors of viral proteases

Proteases catalyze their own release and liberate other nonstructural proteins (Nsps) from the polyprotein, building a replicase–transcriptase complex (RTC) that is vital for viral transcription and replication [36]. Both Mpro and PLpro are essential for viral replication, making them attractive targets for drug development. The high level of structural conservation among the Mpros of 12 different CoVs could give a chance to design a pan-coronavirus inhibitor of viral proteases, and target-specific inhibitors also could be developed for the SARSCoV-2 Mpro [61]. In addition, Mpro has no human homolog. The above features make it an attractive drug target against CoVs.

### 3.1. Lopinavir/ritonavir

Lopinavir and ritonavir were the first drugs used in the clinical trials to treat COVID-19 targeting M^pro^. Although lopinavir/ritonavir showed inhibitor effects against SARS-CoV-2 in Vero E6 cells with an estimated EC50 of 26.63 μM [62], in three RCTs among hospitalized patients with COVID-19, treatment with lopinavir/ritonavir has failed to show any beneficial effect on mortality or need for invasive mechanical ventilation or 28-day hospital discharge rates [63, 64, 65]. Whether lopinavir-ritonavir has a role in outpatients with the nonsevere disease is uncertain, it should be used in outpatients only in the context of a clinical trial. Lopinavir-ritonavir is highly protein-bound and does not appear to achieve plasma levels close to the defined EC50 [66, 67]. 

Current guidelines are against the use of the combination lopinavir/ritonavir among hospitalized patients with COVID-19^6^. 

### 3.2. GC-376

A number of Mpro inhibitors are in different stages of preclinical and clinical development. GC-376 is a representative Mpro inhibitor that has shown antiviral activity against feline infectious peritonitis (FIP) CoV in experimentally infected cats. GC-376 showed promising antiviral activity against the SARS-CoV-2 virus with an EC50 of 3.37 µM [68] and was found to increase survival in MERS-CoV–infected mice [69]. 

Antiviral activity of GC-376 was also shown in SARS-CoV-2-infected K18hACE2 mice, although it was modest consisted with its moderate in vitro antiviral activity. In comparison, a GC-376 analog 6j was recently reported to improve survival in MERS CoV-infected mice, and the EC50 value of the in vitro cellular antiviral activity of 6j against MERS-CoV was 0.04±0.02 µM. This result suggests that in vitro cellular antiviral activity of GC-376 against SARS-CoV-2 needs to be improved by 10–100-fold to achieve desired in vivo antiviral efficacy [70]. 

### 3.3. PF-07304814 and PF-07321332 

PF-00835321 and its novel designed phosphate prodrug PF-07304814 are potent inhibitors of the coronavirus family Mpro in vitro, with selectivity over human host protease targets. 

It was first developed for SARS in 2003, but there wasn’t any chance to fully optimize it for clinical use because of the end of the epidemic. When SARS-CoV-2 came along and genomic analyses revealed that the virus’s Mpro protein was almost identical to that from the original SARS pathogen, studies were started for COVID-19, and preclinical studies revealed that PF-00835231 exhibits potent in vitro antiviral activity against SARS-CoV-2 with suitable pharmaceutical properties as a single agent and is additive/synergistic in combination with remdesivir [71] Boras B, Jones RM, Anson BJ, Arenson D, Aschenbrenner L, et al. Discovery of a novel inhibitor of coronavirus 3CL protease as a clinical candidate for the potential treatment of COVID-19. bioRxiv [Preprint]. 2020:2020.09.12.293498. doi: 10.1101/2020.09.12.293498; [accessed 16 June 2021].. 

The oral form of intravenously administered PF-07304814 was also developed, which is named PF-07321332. PF-07321332 may have inhibitory effects against not only viral proteases but also host proteases, which could be resulted in synergistic effects with superior clinical efficacy Kumar A. Network Pharmacology analysis of orally bioavailable SARS-COV2 protease inhibitor shows synergistic targets to improve clinical eficacy. https://www.researchsquare.com/article/rs-513595/v1[accessed 16 June 2021].. 

The oral bioavailability of PF-07321332 will be a major advantage in the clinical management of COVID-19. PF-07304814 (IV) and PF-07321332 (oral) entered phase 1 clinical trials last September in hospitalized COVID-19 patients and healthy participants, respectively (NCT04535167, NCT04756531) [2]. 

## 4. Inhibitors of viral RNA

### 4.1. Viral RdRp inhibitors

#### 4.1.1.Favipiravir

Being a guanosin purin nucleotide analogue, favipiravir (T-705; 6-fluoro-3-hydroxypyrazine-2- carboxamine) is metabolized intracellularly into its active ribofuranosyl 5’-triphosphate (favipiravir-RTP) metabolite. Favipiravir-RTP is a potent inhibitor of RdRp of RNA viruses and induces lethal mutagenesis. It has shown broad-spectrum activity against variety of RNA viruses including influenza, arenaviruses, bunyaviruses, and flaviviruses. The only oral form of favipiravir is available and after taking orally, it is metabolized by aldehyde oxidase in the liver and excreted renally. Favipiravir is approved for the treatment of epidemic new influenza viruses in Japan. It has been used as an anti-COVID-19 drug in so many countries, including Turkey, India, Russia with a EUA since the beginning of the Pandemic. 

It was shown to be active against SARS-CoV-2 in vitro in Vero cells with (EC50) ranging from 62 to > 500 µM (10 to > 78 µg/mL). Mechanism of actions of favipiravir against SARS-CoV-2 was shown to be through a combination of chain termination, reduced RNA synthesis and lethal mutagenesis in vitro. As it exerts stronger antiviral effect after reaching steady state serum concentration which takes 1 to 2 days, earlier treatment is expected to be more effective. Embryonic lethality and teratogenicity have been shown in animals, as a result, it should not be used in pregnant and nursing women. Also, abstinence or contraception should be recommended 7 days after the end of the favipiravir treatment for males.

Recommended dosage for influenza is 1600 mg, twice a day for the first day, 600mg twice a day for the day 2 to 5 [72–76]. But the optimal dose of favipiravir for the treatment of COVID-19 has yet to be determined. In a hamster model of SARS-CoV-2 infection, while low dose of favipiravir resulted in no reduction in virus levels, high doses of it significantly reduced infectious virus titers in the lungs, markedly improved lung histopathology and decreased virus transmission by direct contact. In that study, the decrease of infectious virus titers was more than the decrease of viral RNA copies. This discrepancy was shown to be due to the mutagenic effect of favipiravir, as the mean number of mutations in the viral RNA increased by a factor of more than three upon favipiravir treatment. Plasma trough concentrations to potently block virus infection as measured in the infected hamsters may be achievable in humans treated with favipiravir [77]. In a clinical trial in Ebola virus-infected patients, a favipiravir dosing scheme of 6.000 mg on day 0, followed by 1.200 mg BID for 9 days, resulted in a median plasma trough concentration of 25.9 μg/mL at day 4 [78]. A favipiravir dosing of 1.800 mg twice on the first day, followed by 800 mg orally twice a day achieves plasma concentration of approximately 60μg/mL and higher in healthy individuals [79]. But it has been reported that the trough concentrations in critically ill COVID-19 patients are lower than those in healthy persons and do not reach the in vitro obtained EC50 values against SARS-CoV-2 [80, 81]. 

In another hamster model study, high doses of favipiravir were found to be associated with antiviral activity against SARS-CoV-2 infection, and the better antiviral efficacy was observed using a preventive strategy, suggesting that favipiravir could be more appropriate for a prophylactic use. When treatment is initiated before or simultaneously to infection, favipiravir has a strong dose effect, leading to reduction of infectious titers in lungs and clinical alleviation of the disease. But the highest dose of favipiravir tested is associated with signs of toxicity in animals. Thereby, pharmacokinetic and tolerance studies are required to determine whether similar effects can be safely achieved in humans. Lung penetration of favipiravir in hamsters has been shown to be efficient, resulting in lung/plasma ratios of 35 to 44% after repeated dosing. However, it is not known whether the lung penetration in humans is similar to that in hamsters [82].

In an exploratory RCT, favipiravir didn’t demonstrate significant in vitro antiviral activity up to 100 μM and under a dosage of 1600 mg or 2200mg loading and 3X600 mg/day maintenance, the insufficient exposure of favipiravir resulted in no additional antiviral benefit among patients with COVID-19 [83]. Low loading dose of favipiravir (≤45 mg/kg/day) was identified as a poor prognostic factor for early clinical improvement in a clinical trial from Tailand Rattanaumpawan P,  Jirajariyavej S,  Lerdlamyong K,  Palavutitotai N, Saiyarin J. Real-world Experience with Favipiravir for Treatment of COVID-19 in Thailand: Results from a Multicenter Observational Study. medRxiv 2020; preprint doi: https://doi.org/10.1101/2020.06.24.20133249[accessed 16 June 2021]. as a result administration of appropriate loading and maintenance doses of favipiravir are of utmost importance.

The results of clinical studies of favipiravir for the treatment of COVID-19 were conflicting. In an earlier metaanalysis, the pooled analysis of five studies showed that favipiravir was associated with a higher clinical improvement rate than control group, but the difference was not statistically significant (odds ratio [OR], 1.54; 95% CI, 0.78–3.04); viral clearance rate at day 4–5, 7-8 and 10–12 was also not different between favipiravir and comparator, additionally the risk of adverse event was similar between groups [84].

But in another recent metaanalysis of 9 studies comparing the efficacy of favipiravir with other control groups revealed a significant clinical improvement in the favipiravir group versus the control group during seven days after hospitalization (RR = 1.24, 95% CI: 1.09–1.41; p = 0.001). Although viral clearance was more in 14 days after hospitalization in favipiravir group, this finding was not statistically significant (RR = 1.11, 95% CI:0.98–1.25; p = 0.094). The need for supplemental oxygen therapy was found to be 7% less in the favipiravir group than the control group, (RR = 0.93, 95% CI: 0.67–1.28; p = 0.664). ICU transfer and adverse events were not statistically different between the groups. Finally, the mortality rate was found to be 30% less in the favipiravir group, but, again, this finding was not statistically significant [85]. 

In another recent metaanalysi, among 5 studies included a comparator group, the favipiravir group showed significantly better viral clearance on day 7 after the initiation of treatment (odds ratio [OR] = 2.49, 95% confidence interval [CI] = 1.19–5.22), but there was no difference on day 14 (OR = 2.19, 95% CI = 0.69–6.95). Clinical improvement was significantly better in the favipiravir group on days 7 and 14 with an OR of 1.60, 95% CI = 1.03–2.49, and 3.03, 95% CI = 1.17–7.80, respectively [86]. 

Unfortunately, all of the studies included in those metaanalyses have varied designs and comparators and the low number of patients; as a result, their findings are based on the analysis of data with high heterogenicity. That’s why further large scale randomized, double blind trials are needed to be done to define the role of favipiravir in the treatment of COVID-19. 

In the single-blinded phase III RCT trial of Japan FUJIFILM Toyama Chemical Co., Ltd. Anti- influenza drug Avigan® tablet meets primary endpoint in phase III clinical trial in Japan for COVID- 19 patients. , June 5, 2021. Website https://www.fujifilm.com/jp/en/news/hq/5451?_ga=2.10224 8257.19488 31102.1612073055 - 48278 478.16120 73055. [accessed 16 June 2021]., 156 patients with COVID- 19 with nonsevere pneumonia were included, favipiravir met the primary end point, which was time to viral clearance and to alleviation of symptoms. The results were 11.9 days and 14.7 days for the favipiravir and the placebo groups, respectively (P =0.0136). But, after reviewing these data, favipiravir was not approved for the use for COVID-19 by the Japan Pharmaceutical Affairs and Food Sanitation Council (PAFSC) because of the single-blinded design of the study, the uncertain primary outcome and the founded biases between the groups. Approval of favipiravir in Japan would be reevaluated after the results of forthcoming ongoing clinical trials conducted in Kuwait and United States [87].

Double- blind phase-III trial in Kuwait was terminated on January 27, 2021, because interim analysis of the study involving 353 patients hospitalized with moderate to severe COVID- 19, did not show a statistically significant difference for the clinical recovery between favipiravir and placebo (7 versus 8 days, p > 0.05). But, the subgroup analysis of the low-risk study cohort (n = 181) demonstrated a 3 day earlier discharge in the favipiravir group compared to the placebo group (8 days versus 11 days; p = 0.0063) for time to hospital discharge. 

The subgroup analysis data during the initial interim analysis points towards the hypothesis with clinically significant insights from this study that an antiviral drug such as favipiravir may be effective as part of early treatment initiation in COVID-19 patients and not effective in the late-stage hospital treatment for moderate and severe COVID-19 patients Dr. Reddy’s and GRA announce Avigan Pivotal Studies Update Study for hospitalized moderate to severe cases in Kuwait terminated, while study for out-patient mild to moderate cases continues in North America. June 5, 2021 . Website https://www.drreddys.com/media/928938/2021-01-avigan-trial-update_v1.pdf. [accessed 16 June 2021].. Another phase III ongoing pivotal study of favipiravir (PRESCO study) for patients with COVID- 19 with mild to moderate symptoms in the United States are planned to include 826 patients Appili Therapeutics. First patient dosed in Appili therapeutics’ Phase 3 clinical trial of Avigan® Tablets (Favipiravir) for the treatment of COVID-19 in the United States. December 2, 2020. Website https://www.appilitherapeutics.com/newsf eed/First -Patient-Dosed-in-Appili-Therapeutics%E2%80%99-Phase-3-Clinical-Trial-of-Avigan%C2%AE-Tablets-%28Favipiravir%29-for-the-Treatment-of-COVID-19-in-the-United-States. [accessed 16 June 2021].. After interim analysis of 600 participitants on May 17, 2021, the Data and Safety Monitoring Board reported no safety issues; as a result, the study will continue without modification and is expected to be completed in the third quarter of 2021 Appili Therapeutics. Independent Data Safety Monitoring Board Recommends Appili Therapeutics Complete Its Phase 3 Avigan®/Reeqonus™ Trial for Mild-to-Moderate COVID-19 Patients. Website https://finance.yahoo.com/news/independent-data-safety-monitoring-board-123100244.html?guccounter=1&guce_referrer=aHR0cHM6Ly93d3cucmVkZGl0LmNvbS8&guce_referrer_sig=AQAAAB2mYpnc8_rgfPV4dOAEl0acVlYeITGio0aVEK7BI5OSVuUDLqEwdu8MeLP1VkSsfE0y6yJIygRaXOtI2mym4IdBKbGGG1K-DoPtyZfoDqRmh35fObEF3zp-2tM7q4xeoFkCm0QeNupCM06dMubZUhzwlFz8L2vL2orEVfLjxfv4. [accessed 16 June 2021].. Favipiravir is also being investigated as a treatment option for COVID-19 among outpatients in an arm of PRINCIPLE (The Platform Randomised trial of Interventions against COVID-19 In older) RCT’s in the UK. People aged 50 to 64 with certain underlying health conditions or shortness of breath from COVID-19, or aged over 65, are eligible to join the favipiravir arm of PRINCIPLE within the first 14 days of experiencing COVID-19 symptoms PRINCIPLE. Favipiravir to be investigated as a possible COVID-19 treatment for at-home recovery in the PRINCIPLE trial. 8 April 2021. Website https://www.principletrial.org/news/favipiravir-to-be-investigated-as-a-possible-covid-19-treatment-for-at-home-recovery-in-the-principle-trial. [accessed 16 June 2021].. 

With the results of these final studies, the effectiveness of favipiravir in the treatment of COVID-19 will be defined more clearly by the end of 2021. 

Additional clinical studies are needed to define the effectiveness of favipiravir at different dosages or at different stages of COVID-19 and PK of favipiravir should be thoroughly evaluated in those patients. 

#### 4.1.2.Remdesivir 

Remdesivir is a prodrug of adenosine nucleotide analog that is intracellularly metabolized to an analog of adenosine triphosphate that inhibits viral RdRp. It was originally developed for the treatment of Ebola and Marburg virus infections. Remdesivir has broad spectrum activity against members of several virus families, including filoviruses (e.g., Ebola) and coronaviruses (e.g., SARS-CoV and MERS-CoV) and has shown prophylactic and therapeutic efficacy in nonclinical models of these coronaviruses. In vitro testing has also shown that remdesivir has an activity against coronoviruses including SARS-CoV, MERS-CoV and SARS-CoV-2 with an EC50 value of 0.09 μM, 0.18 μM and 0.77 μM, respectively [88-90]. Being a monophosphoramidate prodrug, remdesivir is metabolized to its active form, GS-441524 and is recognized as a substrate by RdRp and causing premature termination of viral RNA transcription. It shows resistance to exonucleases of coronaviruses [88] 

In a macaque model, unlike vehicle-treated animals, macaques treated early with remdesivir did not show signs of respiratory disease; they also showed reduced pulmonary infiltrates on radiographs and reduced virus titres in bronchoalveolar lavages 12 h after the first dose. But virus shedding from the upper respiratory tract was not reduced by remdesivir treatment [91].

A metaanalysis of 4 RCTs with 7333 participants about the effectiveness of remdesivir on COVID-19 [65, 92, 93, 94] found no evidence that remdesivir improved outcomes that matter to patients such as reduced mortality, need for mechanical ventilation, time to clinical improvement and others. However, the low certainty evidence for these outcomes, especially mortality, does not prove that remdesivir is ineffective; rather, there is insufficient evidence to confirm that it does improve patient-important outcomes with an odds ratio (95%CI) 0.9 (CI 95% 0.7–1.12); 0.89 (CI 95% 0.76–1.03); 1.06 (CI 95% 0.06–17.56) and 1 (CI 95% 0.37–3.83), for 28 days mortality; mechanical ventilation; viral clearance at day 7; serious adverse events leading to discontinuation respectively^22^. As a result, WHO suggests against administering remdesivir in addition to usual care among hospitalized patients with COVID-19 infection, regardless of the severity of the disease WHO, Therapeutics and COVID-19: living guideline – World Health Organization (WHO), 31 March 2021. [accessed 16 June 2021].. 

But IDSA suggests treatment with 5 days of remdesivir only for patients with COVID-19 on supplemental oxygen but not on mechanical ventilation or ECMO or, without the need for supplemental oxygen and oxygen saturation >94% on room air, as a conditional recommendation and very low certainty of evidence^5^. In the pooled analysis of RCT’s about the effectiveness of remdesivir for patients with COVID-19 on supplemental oxygen but not on mechanical ventilation or ECMO [65, 92, 93], Infectious Disease Society of America (IDSA) also could not find any statistically significant mortality (RR: 0.92; 95% CI: 0.77, 1.10) or clinical improvement benefit at day 28, but they find that patients receiving treatment with remdesivir trend toward greater clinical improvement at 28 days than patients not receiving remdesivir (RR: 1.13; 95% CI 0.91–1.41). 

In addition, based on a post-hoc analysis of patients with severe COVID-19, they find that receiving treatment with remdesivir had a shorter median time to recovery (median 11 versus 18 days; Rate ratio: 1.31; 95% CI: 1.12, 1.52) and decreased need for mechanical ventilation (RR: 0.57; 95% CI: 0.42, 0.79)^6^.

Current findings of the effectiveness of remdesivir for the treatment of patients for COVID-19 are conflicting, additionally the IV route of administration of remdesivir prevents its widespread usage for the treatment of COVID-19. 

#### 4.1.3. Molnupiravir

Molnupiravir (MK-4482/EIDD-2801) is the orally available pro-drug of the nucleoside analogue N^4^-hydroxycytidine (NHC), which has broad-spectrum anti-RNA virus activity including influenza ebola, CoV, respiratory syncytial virus, and Venezuelan equine encephalitis virus (VEEV). Serial passaging in the presence of NHC led to low level resistance for VEEV but not RSV, influenza A virus, and bovine viral diarrhea virus, thus, indicating a high resistance barrier [95]. NHC was found to be potently antiviral with an IC50 of 0.08 mM and IC50 of 0.024 mM against SARS-CoV-2 in the Calu-3 cells and human airway epithelial cells, respectively. It was also shown to has a broad-spectrum antiviral activity against MERS-CoV, SARS-CoV, and related zoonotic group 2b or 2c bat-CoVs, as well as increased potency against a CoVs bearing resistance mutations to the nucleoside analog inhibitor remdesivir. Its effect was found to be associated with increased transition mutation frequency in viral but not in host cell RNA. Both prophylactic and therapeutic administration of molnupiravir improved pulmonary function and reduced virus titer and body weight loss in mice infected with SARS-Cov-2. The potency of molnupiravir against multiple CoVs and oral bioavailability highlights its potential utility as an effective antiviral against SARS-CoV-2 and other future zoonotic CoVs [96]. 

In a ferret model, therapeutic treatment of infected animals with twice-daily molnupiravir significantly reduced upper respiratory tract SARS-CoV-2 load and completely suppressed spread to untreated contact animals, and it was seen that earlier treatment of ferrets with molnupiravir was more beneficial [97].

Single and multiple doses of molnupiravir were evaluated in a phase I randomized, double-blind, placebo-controlled study in healthy volunteers, and demonstrated good tolerability and dose-proportional pharmacokinetics with relatively low variability following administration to healthy volunteers at clinically relevant doses Painter WP, Holman W, Bush JA, Almazedi F, Malik H, et al. Human Safety, Tolerability, and Pharmacokinetics of a Novel Broad-Spectrum Oral Antiviral Compound, Molnupiravir, with Activity Against SARS-CoV-2. medRxiv preprint 2020; doi: https://doi.org/10.1101/2020.12.10.20235747 [accessed 16 June 2021]..

Based on a planned interim analysis of data from the Phase II/III trials, the decision has been made to proceed with the Phase 3 portion of the study in outpatients (MOVeOUT), but not in hospitalized patients (MOVeIN) with COVID-1. This is because the data from hospitalized patients indicate that molnupiravir is unlikely to demonstrate a clinical benefit in hospitalized patients, who generally had a longer duration of symptoms prior to study entry. In an interim analysis of MOVe-OUT study, the percentage of patients who were hospitalized and/or died was lower in the molnupiravir-treated groups versus the placebo arm, but the difference was not statistically significant. Nasopharyngeal SARS-CoV-2 clearance rate was higher in molnupiravir treated patients than in placebo group (6/25 versus 0/47) at day 5 of treatment (p = 0.001). These differences in virology endpoints were more pronounced in participants enrolled <5 days following symptom onset. After enrolment of 1850 patients, the final results of MOVeOUT study are expected by Agust, 2021 Ridgeback Biotherapeutics and Merck Announce Preliminary Findings from a Phase 2a Trial of Investigational COVID-19 Therapeutic Molnupiravir.Website https://www.businesswire.com/news/home/20210305005610/en/. [accessed 16 June 2021].. 

In addition to Phase-3 RCT, it is planned to initiate a clinical trial to evaluate molnupiravir for postexposure prophylaxis in the second half of 2021.

#### 4.1.4. AT-527

AT-527 is an orally available prodrug of a guanosine nucleotide analog that has demonstrated potent in vitro activity against clinical isolates of hepatitis C virus (HCV) and found to be effective in the treatment of HCV-infected patients [98]. AT-511, the free base of AT-527 also has potent antiviral activity in vitro against several human coronaviruses, including SARS-CoV-2. The active triphosphate metabolite of AT-527, AT-9010, which cannot penetrate to cell membranes and is formed only after intracellular delivery of the prodrug, is produced in substantial amounts in primary human cells of the respiratory tract incubated with AT-511. In normal human airway epithelial cells, the concentration of AT-511 required to inhibit replication of SARS-CoV-2 by 90% (EC90) was 0.47mM, very similar to its EC90 against human coronavirus (HCoV)-229E, HCoV-OC43, and SARS-CoV in Huh-7 cells. After oral dosing, the predicted concentration of the active metabolite in pulmonary tissue suggests that AT-527 may be an effective treatment option for individuals infected with COVID-19 [99]. AT-9010 simultaneously binds to both NIRAN and RdRp active site of nsp12 and blocking their respective activities, which should attenuate the chance of resistance mutations Shannon A , Fattorini V, Sama B , Selisko B , Feracci M , et al. Protein-primed RNA synthesis in SARS-CoVs and structural basis for inhibition by AT-527. bioRxiv preprint 2021; doi: https://doi.org/10.1101/2021.03.23.436564.. 

The ongoing phase II and III clinical trials (NCT04709835, NCT04396106, NCT04889040) of AT-527 for the treatment of both out and in patients with COVID-19 are expected to be completed at the end of 2021. 

### 4.2. Inhibitors of host proteins supporting viral RNA synthesis 

#### 4.2.1. Inhibitors of inosine monophospate dehydrogenase (IMPD): Merimepodib

IMPD is an enzyme responsible for de novo synthesis of guanosine nucleotides that SARS-CoV-2 needs it during transcription in the host cell. Merimepodib noncompetitively inhibits IMPD and, inhibited SARS-CoV-2 with concentrations as low as 3.3 μM in Vero cells [100]. But a phase 2 RCT of merimepodib in combination with remdesivir in adult patients with severe COVID-19 was terminated because of the failure to meet the primary endpoint (NCT04410354). 

#### 4.2.2. Inhibitor of dihydroorarate dehydrogenase: PTC299 

Human dihydroorotate dehydrogenase (DHODH) is a key enzyme of pyrimidine de novo biosynthesis pathway. SARS-CoV-2 hijacks that pathway to replicate. A DHODH inhibitor, PTC299 which was originally designed as oral drug for acute myeloid leukemia- manifested robustly, dose-dependent, inhibition of SARS CoV-2 replication with an EC_50_ range of 2.0 to 31.6 nM on Vero-cells [28, 101].

### 4.3. Cas13a

A gene-editing enzyme Cas 13a could seek out and cuts RNA in to pieces. A Cas13a enzyme targeted to 2 SARS-CoV-2 genes encoding the RdRp and the N protein has been developed and was reduced SARS-CoV-2 replication and reduced symptoms in a hamsters models of the COVID-19 [102]. 

## 5. Inhibitors of host proteins supporting viral protein synthesis (Inhibitors of eEF1A (plitidepsin); inhibitors of ER chaperon protein (S1R) (fluvoxamin))

There are important advantages of targeting the host proteins, which include creating a higher barrier for viruses to develop resistance and broader protection against diverse viral strains [103].

Among numerous host proteins that play a role in the viral life cycle of SARS-CoV-2, those targeted the eukaryotic translation machinery demonstrated particularly potent antiviral activities. Zotatafin, an inhibitor of eukaryotic translation initiation factor 4A (eIF4A); plitidepsin, an inhibitor of eukaryotic translation elongation factor 1a (eEF1A), and ternatin-4, also an inhibitor of eEF1A show antiviral activity against SARS-CoV-2. Among those plitidepsin is the most effective. 

### 5.1. Plitidepsin

Plitidepsin was found to be more potent than remdesivir tested in the human pneumocyte-like cells line by a factor of 27.5 and treatment of mice with plitidepsin reduced viral lung titers and lung pathology upon infection to a similar degree as remdesivir [104]. There are concerns about the use of translation inhibitors, because of potential toxicities arising from the systemic inhibition of host translation. But plitidepsin was evaluated in many cancer clinical trials and the safety profile is well established [105].

A phase I/II clinical study of plitidepsin for the treatment of COVID-19 has been completed (NCT04382066), and zotatifin, is also in clinical trials for the treatment of COVID-19 (NCT04632381). Results of plitidepsin’s Phase I/II has not been published yet but promising results were released [105]. The ongoing phase III clinical trial (NCT04784559) will show whether plitidepsin is effective for COVID-19. The study is expected to be completed in November, 2021. 

### 5.2. Fluvoxamin

It was previously suggested that antidepressants may be associated with decreased plasma levels of some inflammatory mediators [106]. A selective serotonin reuptake inhibitor, fluoxetine was shown to have antiviral activity against SARS-CoV-2 with an EC50 level off 0.387 µg/ml in Vero and Huh7 cell and to inhibit SARS‑CoV‑2 in human lung tissue [107]. 

In an observational study, antidepressant use was found to be associated with reduced risk of intubation and death in patients hospitalized for COVID-19 [108]. 

In a preliminary RCT of 52 COVID-19 patients, clinical deterioration rate was lower in the fluvoxamine group than in the placebo group (absolute difference, 8.7% [95% CI, 1.8%–16.4%], p = 0.009) [109]. Potential mechanisms suggested for the effect of fluvoxamine in COVID-19 were ER chaperon protein sigma-1 receptor (S1R) agonism and functional inhibition of acid sphingomyelinase activity (FIASMA), which were shown to prevent the infection of epithelial cells with SARS-CoV-2 [110].

The larger randomized trials with more definitive outcome measures are required to define the role of fluvoxamine in the treatment of COVID-19. 

## 6. Inhibitors of viral immunomodulation (Inhibitors of host importin α/β (ivermectin)

### 6.1. Ivermectin

Ivermectin is an FDA-approved broad-spectrum antiparasitic agent, it has shown to have antiviral activity against a broad range of viruses in vitro in recent years. Ivermectin has been shown to inhibit the nuclear import of host and viral proteins. In an in vitro study, ivermectin was found to be an inhibitor of the SARS-CoV-2, with a single addition to Vero-hSLAM cells 2 h postinfection with SARS-CoV-2 able to effect ~5000-fold reduction in viral RNA at 48 h. Authors hypothesize that this was likely through inhibiting IMPα/β1- mediated nuclear import of viral proteins (as shown for other RNA viruses), and this inhibition disrupts the immune evasion mechanism of the virus [111]. But, after analyzing published pharmacokinetic data from clinically relevant and excessive dosing studies, it was concluded that the SARS-CoV-2 inhibitory concentrations are not likely to be attainable in humans [112, 113]. 

16 RCTs with 2407 patients having either severe or non-severe COVID-19 were evaluated in a systematic review of WHO, and it was seen that only five studies directly compared ivermectin with standard care and reported mortality. While pooled estimate of those five RCTs suggests a reduction in mortality with ivermectin, this effect was not apparent if only the trials at low risk of bias were considered. In addition to concerns about the risk of bias, serious concerns related to imprecision for the outcome of mortality were noticed. Furthermore, the evidence informing this comparison was from multiple small trials, adding to the risk of unrecognized imbalances in study arms. Given the strong likelihood that chance may be playing a role in the observed findings, the WHO panel suggest that there was very serious imprecision, further lowering the overall certainty in findings. They concluded that there was insufficient evidence about the effectiveness of ivermectin in treating Covid-19; there was a large degree of uncertainty in the evidence about ivermectin on mortality, need for mechanical ventilation, need for hospital admission, time to clinical improvement, and other patient-important outcomes, and there was potential for harm with an increased risk of adverse events leading to study drug discontinuation. As a result, WHO recommends not to use ivermectin in patients with Covid-19 except in the context of a clinical trial, regardless of disease severity or duration of symptoms^21^. 

In line with the WHO’s analysis and recommendation, IDSA also suggested against ivermectin for the treatment of hospitalized patients with COVID-19, unless in the context of a clinical trial. They also suggest against ivermectin for the treatment of outpatients with COVID-19, unless in the context of a clinical trial and concluded that well-designed, adequately powered, and well-executed clinical trials are needed to inform decisions on treating COVID-19 with ivermectin ^5^. 

One metaanalysis about the efficacy of ivermectin in prophylaxis of COVID-19 included 3 studies; 2 with ivermectin alone (n = 540), and 1 with ivermectin combined with iota-carrageenan (n = 234), all compared with standard care or placebo. Again, serious risk of bias and very serious imprecision, and, thus, very low certainty of evidence was determined about the effects of ivermectin combined with iota-carrageenan on laboratory confirmed Covid-19 (52 fewer per 1000, 58 fewer to 37 fewer), ivermectin alone on laboratory confirmed infection (50 fewer per 1000, 59 fewer to 16 fewer) and suspected, probable, or laboratory confirmed infection (159 fewer per 1000, 165 fewer to 144 fewer). They concluded that because of serious risk of bias and very serious imprecision, it was highly uncertain whether ivermectin combined with iota-carrageenan and ivermectin alone reduces the risk of SARS-CoV-2 infection [114].

## 7. Agents supporting host natural immunity

### 7.1. Interferons

All viruses trigger an antiviral response that relies on the immediate production of IFNβ in the host. The binding of IFNβ to its receptor then triggers the production of IFN-α. Both IFNs trigger the expression of hundreds of interferon-stimulated genes (ISGs). If the production of IFNα/β occurs immediately and is intense enough, the infection can be stopped. This is probably what happens for SARS-CoV-2- infected individuals who remain asymptomatic or have mild disease, as in almost all children. However, the virus-induced IFNα/β response may be weak, due to immunosenescence, comorbidities, and anti-IFN mechanisms that most viruses have developed through evolution [7]. Recent studies reveal that IFN dysregulation is key to determine COVID-19 pathogenesis. Inborn errors of TLR3- and IRF7-dependent type I IFN immunity were found to be related to life-threatening COVID-19 pneumonia [115]. Additionally, neutralizing IgG auto-antibodies against IFN-α2 or IFN-ω, or both were determined in 10% of patients with life-threatening COVID-19 pneumonia, and all of these patients tested had low or undetectable serum IFN- α levels during acute disease [116]. In situations of inefficient IFN response, the virus replicates and this triggers a second inflammatory/ immune response, which may become explosive and potentially result in a cytokine storm and ARDS. Prophylactic administration of IFNs at the early stage may elicit an autonomous antiviral state and prevent COVID-19 progression [117]. But, contrary to this hypothesis, in SOLIDARITY clinical trial, death occurred in 243 of 2050 patients receiving interferon and in 216 of 2050 receiving control (rate ratio, 1.16; 95% CI, 0.96 to 1.39; P=0.11), and IFN did not reduce mortality, overall or in any subgroup, or reduced initiation of ventilation or hospitalization duration [65]. Several factors seem to affect IFNs efficacy, including disease severity and treatment onset time. As nearly ¾ of patients were on supplemental oxygen at the time of randomization in the SOLIDARITY trial, IFN treatment may be delayed for this group of patients. 

In another RCT in adults with confirmed COVID-19, clinical recovery rate was greater in patients who received inhaled nebulised interferon beta-1a (SNG001) placebo (odds ratio 2·32 [95% CI 1·07–5·04]; p = 0·033) [118]. 

In a three-armed, RCT of IFNβ1a and IFNβ1b in patients with confirmed SARS-CoV-2 infection, as compared with the standard care, the benefit of a significant reduction in clinical improvement time was observed only in the IFNβ1a arm. This finding needs further confirmation in larger studies [119]. 

Finally, in a metaanalysis of five RCT’s about the effectiveness of IFN- β for treatment of COVID-19, the average mortality rate was reported as 6.195% and 18.02% in intervention and control groups, respectively. Likewise, the median days of hospitalization were found to be lower in the intervention group (9 days) than the control group (12.25 days), and IFN-β was found to increase the overall discharge rate (RR = 3.05; 95% CI: 1.09-5.01) [120]. 

In conclusion, IFN β could have a role in the treatment of COVID-19, especially if started earlier in the course of the disease, but further RCTs including a larger number of patients are needed. 

## 8. Conclusion

Taking into account the results of all the available laboratory and clinical trials on the subject, monoclonal antibodies seem to be the most effective treatments for COVID-19 at the moment and high-titer convalescent plasma also could be effective when administered during the early phase of the disease. As lopinavir/ritonavir, hydroxychloroquine, merimepodib, and umifenovir were found to be ineffective in RCTs, they should not be used for the treatment of COVID-19. Additional clinical trials are needed to define the role of remdesivir, favipiravir, interferons, ivermectin, dutasteride, proxulutamide, fluvoxamine, bromhexine, nitazoxanide, and niclosamid in the treatment of COVID-19. Finally, the results of phase trials are waited to learn whether or not the newer agents such as molnupiravir, PF-07321332, PF-07304814, plitidepsin, and AT-527 are effective in the treatment of COVID-19. 
